# Glucagon receptor signaling at white adipose tissue does not regulate lipolysis

**DOI:** 10.1152/ajpendo.00078.2022

**Published:** 2022-08-24

**Authors:** Anastasiia Vasileva, Tyler Marx, Jacqueline L. Beaudry, Jennifer H. Stern

**Affiliations:** ^1^Division of Endocrinology, University of Arizona College of Medicine, Tucson, Arizona; ^2^Department of Nutritional Sciences, University of Toronto, Toronto, Ontario, Canada

**Keywords:** fasting, glucagon, lipolysis, type 2 diabetes, white adipose tissue

## Abstract

Although the physiological role of glucagon receptor signaling in the liver is well defined, the impact of glucagon receptor (Gcgr) signaling on white adipose tissue (WAT) continues to be debated. Although numerous studies propose that glucagon stimulates WAT lipolysis, we lack evidence that physiological concentrations of glucagon regulate WAT lipolysis. In turn, we performed studies in both wild-type and WAT *Gcgr* knockout mice to determine if glucagon regulates lipolysis at WAT in the mouse. We assessed the effects of fasting and acute exogenous glucagon administration in wild-type C57BL/6J and *Gcgr*^Adipocyte+/+^ versus *Gcgr*^Adipocyte−/−^ mice. Using an ex vivo lipolysis protocol, we further examined the direct effects of glucagon on physiologically (fasted) and pharmacologically stimulated lipolysis. We found that adipocyte *Gcgr* expression did not affect fasting-induced lipolysis or hepatic lipid accumulation in lean or diet-induced obese (DIO) mice. Acute glucagon administration did not affect serum nonesterified fatty acids (NEFA), leptin, or adiponectin concentration, but did increase serum glucose and FGF21, regardless of genotype. Glucagon did not affect ex vivo lipolysis in explants from either *Gcgr*^Adipocyte+/+^ or *Gcgr*^Adipocyte−/−^ mice. *Gcgr* expression did not affect fasting-induced or isoproterenol-stimulated lipolysis from WAT explants. Moreover, glucagon receptor signaling at WAT did not affect body weight or glucose homeostasis in lean or DIO mice. Our studies have established that physiological levels of glucagon do not regulate WAT lipolysis, either directly or indirectly. Given that glucagon receptor agonism can improve dyslipidemia and decrease hepatic lipid accumulation, it is critical to understand the tissue-specific effects of glucagon receptor action. Unlike the crucial role of hepatic glucagon receptor signaling in maintaining glucose and lipid homeostasis, we observed no metabolic consequence of WAT glucagon receptor deletion.

**NEW & NOTEWORTHY** It has been postulated that glucagon stimulates lipolysis and fatty acid release from white adipose tissue. We observed no metabolic effects of eliminating or activating glucagon receptor signaling at white adipose tissue.

## Graphical abstract

**Figure:**
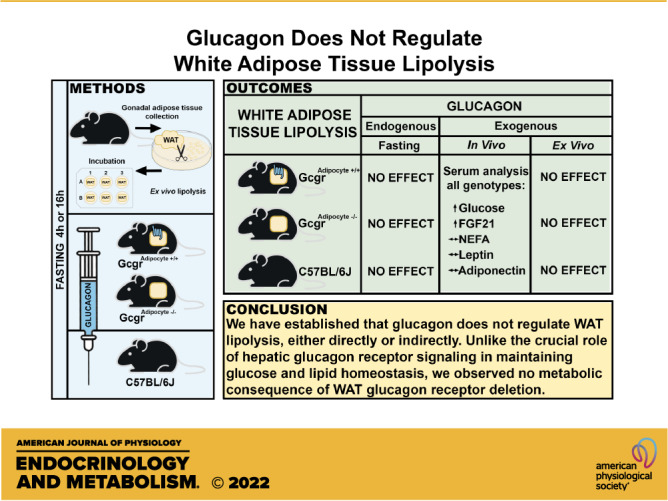

## INTRODUCTION

Glucagon plays a critical role in the maintenance of glucose (1, 2) and lipid (2–5) homeostasis during fasting, primarily by stimulating glycogenolysis, gluconeogenesis, fatty acid oxidation, and inhibiting de novo lipogenesis in the liver. Although the liver is the main site of glucagon action and receptor expression, glucagon receptors have been detected to a far lesser extent in mouse (6), rat (7), and human (8, 9) white adipose tissue (WAT). Still, others have concluded that glucagon receptor mRNA is undetectable in isolated mature adipocytes (10, 11). Whether glucagon receptor signaling at WAT plays a role in metabolic homeostasis is not clear. Glucagon has been shown to directly activate hormone-sensitive lipase in rat epididymal fat pads (12) and stimulate lipolysis in isolated rat (13–17) and human (9) white adipocytes. Yet, clinical studies demonstrate that physiological levels of glucagon have no effect on adipose tissue lipolysis in people with (18) or without (18–20) diabetes.

Obese, insulin-resistant humans (21–23) and mice (23) hypersecrete glucagon in the fed state, exacerbating hyperglycemia and encouraging the development of type 2 diabetes (T2DM). Suppression of glucagon signaling improves glycemic control and decreases hyperglycemia in people with insulin resistance (24, 25). Accordingly, the development of glucagon receptor antagonists and antibodies is currently being pursued as a glucose-lowering therapeutic for patients with T2DM (26–28). Suppression of glucagon signaling is similarly effective at improving glycemic control in hyperinsulinemic/insulin-resistant mice (29, 30). Accordingly, the mouse is an informative model to assess the potential beneficial responses to glucagon receptor manipulation. Despite improving the regulation of blood glucose, reported increases in hepatic triglyceride content, plasma total cholesterol (31), and liver enzymes (31, 32) have led to questions regarding the safety and efficacy of such therapeutics. Global glucagon receptor knockout increases fasting serum nonesterified fatty acid (NEFA) and triglyceride (TAG) concentrations, and hepatic TAG secretion in mice (4). Wild-type mice treated with a single dose of a glucagon receptor antagonist and global glucagon receptor knockout mice display impaired triglyceride clearance during an oral lipid clearance challenge. Treatment with a long-acting glucagon analog decreases plasma triglyceride, suggesting that both chronic and acute glucagon signaling is essential in promoting intestinal lipid clearance and hepatic lipid metabolism (11). Thus, the mouse is also an informative preclinical model to assess the potential negative consequences of glucagon receptor antagonism.

The deleterious effects of glucagon receptor antagonism on lipid homeostasis may be due to a lack of glucagon receptor signaling at the hepatocyte or the adipocyte. The regulation of lipid metabolism in both the liver and adipose tissue is critical in the maintenance of whole body lipid homeostasis. In the liver, glucagon inhibits de novo lipogenesis and stimulates triglyceride hydrolysis and beta-oxidation (3, 4). Glucagon decreases hepatic triglyceride secretion (4) and chronic glucagon administration decreases serum cholesterol (33, 34). Tight regulation of adipose tissue lipolysis is equally a critical factor in the maintenance of lipid homeostasis. For example, in response to an extended fast, an increase in WAT lipolysis increases hepatic triglyceride accumulation (35). Because the amount of fatty acids taken up by the liver exceeds the liver’s capacity to catabolize this lipid, hepatic steatosis ensues (36). Thus, dysregulation of lipolysis at adipose tissue has a profound impact on hepatic lipid metabolism. Suppression of WAT lipolysis decreases hepatic lipid accumulation (37), whereas elimination of insulin-mediated suppression of WAT lipolysis leads to increased hepatic lipid accumulation (38, 39), predisposing to hepatic insulin resistance and the development of liver disease.

We set out to understand if glucagon signaling at WAT is involved in the regulation of WAT lipolysis and potential alterations in hepatic lipid concentration. Given that efforts to develop pharmacological inhibitors of glucagon receptor signaling as a therapy for hyperglycemia in T2DM continue (26, 27, 40), it is critical to understand the potential consequences of blocking glucagon receptor signaling at the adipocyte.

## MATERIALS AND METHODS

### Mice

All mice were maintained on a 12-h light/12-h dark cycle and housed with three to five mice per cage until 1 wk before study initiations, at which time animals were individually housed. Mice were housed with sani-chip bedding (7090 Teklad). All studies were approved by The University of Texas Southwestern and The University of Arizona Institutional Animal Care and Use Committees. All mice were provided ad libitum access to food (2016 Teklad Global 16% Protein Rodent Diet, 12% kcal from fat; Envigo, Indianapolis, IN) and water unless fasting or alternative diets are specified.

Terminal glucagon responsivity studies were performed in male wild-type C57BL/6J mice (Strain No. 000664; RRID:IMSR_JAX:000664) that were bred in-house at UT Southwestern Medical Center. Ex vivo lipolysis assays performed in wild-type male C57BL/6J mice were purchased from Jackson laboratories (Strain No. 000664; RRID:IMSR_JAX:000664).

Adiponectin-rtTA (Apn-rtTA) (41) and floxed *Gcgr* (1) mice were generated as previously described. The TRE-Cre mouse was purchased from Jackson Laboratories (Strain No. 006234; Jackson Laboratories, Bar Harbor, ME) *Gcgr*^Adipocyte−/−^ (adipocyte-specific *Gcgr* knockout; *Apn-rtTA*^+/−^, *TRE-Cre*^+/−^, *Gcgr*^F/F^) mice and littermate *Gcgr* ^Adipocyte+/+^ controls (*Apn-rtTA*^+/−^, *TRE-Cre*^−/−^, *Gcgr*^F/F^) were generated by crossing male *Apn-rtTA*^−/−^, *TRE-Cre*^+/−^, *Gcgr*^F/F^ mice to female *Apn-rtTA*^+/−^, *TRE-Cre*^−/−^, *Gcgr*^F/F^ mice. Mice were fed a standard chow diet (2016 Teklad Global) until ∼8 wk of age, at which point a chow (S4107, Bio-Serv, Flemington, NJ) or high-fat (60% energy from fat; Bio-Serv S5867) diet containing 600 mg/kg doxycycline (DOX) was provided to initiate adipocyte-specific deletion of the glucagon receptor gene. Chow-fed mice were maintained on DOX for 4 wk before the initiation of studies. High-fat-diet-fed mice were maintained on high-fat DOX diet for 14 wk to induce obesity. For all studies, mice were stratified on body mass and randomized to diet (chow or high-fat diet) and/or treatment (saline or glucagon). Investigators were blinded to genotype, treatment, and diet when performing all wet laboratory assays.

### Isolation of Mature Adipocytes and Stromal Vascular Fraction

We isolated stromal vascular and enriched mature adipocyte fractions from gonadal adipose tissue to compare the expression of *Gcgr* in these two cell types. Gonadal white adipose depots were taken from 6-mo-old wild-type C57BL/6J male mice maintained on standard chow (2016 Teklad Global). Mice were euthanized by decapitation after bell jar exposure to isoflurane anesthesia. Gonadal fat pads were immediately excised, minced with scissors and put into 20 mL of 1 mg/mL collagenase type I (Sigma SCR103) in HEPES buffer. Tissue in collagenase buffer was put in a shaking incubator (37°C; 200 RPM) for 90 min and removed every 30 min for mechanical disruption by serological pipette. After 90 min of incubation, the digested tissue was centrifuged at 600 *g* for 5 min to separate floating adipocytes from the pelleted stromal vascular fraction (SVF) (42). Adipocytes were collected from the surface into TRIzol, the remaining buffer was removed, and 1 mL of TRIzol was immediately added to the SVF. Samples were flash frozen in TRIzol and stored at −80°C until RNA isolation.

### Terminal Glucagon Responsivity Tests

To initially examine the effect of exogenous glucagon administration on adipose tissue lipolysis, male wild-type C57BL/6J mice were fasted for either 4 or 16 h before intraperitoneal injection with either saline or glucagon (5 μg/kg body wt; Eli Lily and Company, Indianapolis). Mice were euthanized at either 0 (saline group only), 15, 30, or 60 min after injection by decapitation after bell jar exposure to isoflurane anesthesia. Trunk blood was immediately collected and allowed to clot before centrifugation at 3,000 *g* for 30 min. Serum was collected, aliquoted, and immediately frozen at −80°C. Tissues were collected immediately, rinsed with phosphate-buffered saline, flash-frozen in liquid nitrogen, and stored at −80°C until analysis. Terminal glucagon responsivity tests in *Gcgr* ^Adipocyte+/+^ and *Gcgr*^Adipocyte−/−^ mice were performed identically, with mice euthanized 15 min after injection.

### Crossover Glucagon Responsivity Tests

Male *Gcgr*^Adipocyte+/+^ and *Gcgr*^Adipocyte−/−^ mice were fasted for either 4 or 16 h before intraperitoneal injection with either saline or glucagon (5 μg/kg body wt; Eli Lily and Company, Indianapolis). Thirty minutes after injection, tail blood was collected using a capillary tube and allowed to clot before centrifugation at 3,000 *g* for 30 min. Serum was collected, aliquoted, and immediately frozen at −80°C. Mice were allowed to recover for 3 days before the study was repeated in a crossover fashion.

### Ex Vivo Lipolysis

Ex vivo lipolysis studies were performed in male 16–18-wk-old mice. Mice were fasted for 4 or 16 h before being euthanized by decapitation after bell jar exposure to isoflurane anesthesia. Ex vivo lipolysis was assayed as previously described (43, 44). Lipolysis was assessed in triplicate for each treatment within a mouse. Briefly, gonadal adipose tissue was collected immediately after euthanasia and washed with phosphate-buffered saline before mincing tissue into ∼20 mg pieces. Explants were then incubated for 1 h in Krebs–Ringer buffer (12 mM HEPES, 121 mM NaCl, 4.9 mM KCl, 1.2 mM MgSO_4_, and 0.33 mM CaCl_2_, 3 mM glucose containing 2% fatty acid-free bovine serum albumin) at 37°C, then transferred to Krebs–Ringer buffer containing the following treatments: 10 µM isoproterenol (Sigma, Cat. No. I6504-500MG), 10 µM forskolin (Sigma, Cat. No. F6886-10MG), insulin (20, 200, and 2,000 nM; Fisher, Cat. No. 501657324), glucagon (0.1, 1, 10, and 100 nM; Eli Lilly), or control (Krebs–Ringer only). After 1 h incubation, explants were removed and stored at −80°C until analysis for total protein content. Explants were sonicated in 0.1 M phosphate-buffered saline, pH 7.4 (PBS), then centrifuged for 10 min at 13,000 *g* at 4°C. The top lipid layer was carefully removed before transferring supernatant to a fresh tube. Total protein in the supernatant was assayed using a colorimetric assay (Pierce BCA Protein Assay Kit, Cat. No. 23225). Media was collected and stored at −80°C until analysis for NEFA content via colorimetric assay (999–34691, 995–34791, 991–34891, and 993–35191, Wako Diagnostics). Leptin content in media was analyzed by ELISA (Cat. No. EZML-82K, Millipore Sigma, Danvers, MA).

### Oral Glucose Tolerance Testing via Gavage

After a 4 h fast, we gave individually housed mice an oral gavage of d-glucose (2.5 g/kg; Fisher) and assessed blood glucose by glucometer (9556c, Bayer, Leverkusen, Germany). Blood was collected by tail nick at 0, 15, 30, 60, 90, and 120 min following glucose gavage. Blood for serum insulin (glucose-stimulated insulin secretion) was collected from the tail vein before and 15 min following glucose administration.

### Oral Lipid Clearance Testing via Gavage

After an overnight 16 h fast, olive oil (10 µL/g body wt) (45) was gavaged into individually housed mice. Oral lipid clearance testing began at 9:00 AM. Blood for serum triglyceride was collected via tail vein at 0 min, 30 min, 1, 2, 4, 6, 8, and 10 h following olive oil gavage.

### Tyloxapol Stimulated Triglyceride

After a 4 h fast, individually housed mice were injected with 300 mg/kg Triton WR-1339 (Tyloxapol; Sigma Aldrich) in 0.9% saline via the tail vein (46) to inhibit lipoprotein lipase activity. Studies began at 1:00 PM and blood for serum triglyceride was collected via tail vein at 0, 30, 60, 90, and 120 min following injection.

### Serum Analyses

Commercially available enzyme-linked immunosorbent assays were used to assess hormones in serum and media (Glucagon: Cat. No. 10–1271-01, Mercodia, Uppsala, Sweden; Insulin: Cat. No. 80-INSMSU-E10, Alpco, Salem, NH; FGF21: Cat. No. EZRMFGF21-26K, Millipore Sigma, Danvers, MA; Leptin: Cat. No. EZML-82K, Millipore Sigma, Danvers, MA; and Adiponectin: Cat. No. EZMADP-60K, Millipore Sigma, Danvers, MA). Serum glucose, NEFA, TAG, and total cholesterol concentrations were analyzed by an enzymatic colorimetric assay (Glucose: Cat. No. G7519, Pointe Scientific Inc., Canton MI; NEFA: Cat. No. 999–34691, 995–34791, 991–34891, and 993–35191, Wako Diagnostics USA; TAG: Cat. No. T7531, Pointe Scientific Inc., Canton, MI; and total cholesterol: Cat. No. TR13421, Thermo Scientific, Middletown, VA).

### RNA Isolation and Gene Expression

RNA was isolated using TRIzol Reagent (Thermo Fisher Scientific, Waltham, MA). Phenol was eliminated using the water-saturated butanol and ether method of Krebs, Fischaleck, and Blum (47). Reverse transcription was performed using Verso cDNA synthesis kit (Thermo Scientific, Inc., Waltham, MA), and RT-qPCR was performed using PowerUp SYBR Green Master Mix on the Applied Biosystems QuantStudio 6 Flex Real-Time PCR System (Applied Biosystems, Foster City, CA). LinReg PCR analysis software was used to determine the efficiency of amplification from raw CT data (48). ACTβ served as the reference gene for calculating fold change in gene expression using the efficiency^ΔΔCt^ method (49). *Gcgr* mRNA was detected using TaqMan probes Mm00433546_m1 and m00433550_g1 (ThermoFisher, Waltham, MA). Mouse primer sequences for all other genes for real-time PCR are presented in Table 1.

**Table 1.** List of primer sequences for RT-PCR

Gene	Forward Primer (5′-3′)	Reverse Primer (5′-3′)	Gene ID
*Mouse Actb*	TCGGTGACATCAAAGAGAAG	GATGCCACAGGATTCCATA	11461
*Mouse G6pc*	CGACTCGCTATCTCCAAGTGA	GTTGAACCAGTCTCCGACCA	14377
*Mouse Pepck*	CTGCATAACGGTCTGGACTTC	CAGCAACTGCCCGTACTCC	18534
*Mouse Ppara*	AGAGCCCCATCTGTCCTCTC	ACTGGTAGTCTTGCAAAACCAAA	19013
*Mouse Cpt1a*	CTCCGCCTGAGCCATGAAG	CACCAGTGATGATGCCATTCT	12894

### Hepatic Lipid Content

Livers were powdered with a liquid nitrogen-cooled mortar and pestle to ensure a homogeneous sample. Briefly, 10–20 mg of powdered liver samples were weighed and sonicated in 100-µL PBS. Furthermore, 1 mL of 100% ethanol was added to each sample and vortexed for 20 min then centrifuged at 16,000 *g* at 4°C (50). Supernatant was then transferred to a fresh tube for analysis of liver triglycerides (Cat. No. T7531, Pointe Scientific Inc., Canton, MI). Total hepatic triglyceride content was calculated as milligram per gram of tissue.

### Immunohistochemistry and Imaging of Pancreata

Immediately after mice were euthanized, whole pancreata were collected into 4% paraformaldehyde and fixed for 24 h. Tissues were then transferred to a 50% ethanol solution before paraffin embedding. Paraffin-embedding and tissue-sectioning were performed by the Molecular Pathology Core Facility at University of Texas Southwestern. Slides were then deparaffinized with xylene (3 min incubation) and rehydrated using graded concentrations of water:ethanol (3 min incubations at 0:100, 5:95, 30:70, and 50:50), followed by a 3 min incubation in PBS. Slides were boiled in antigen retrieval solution (10 mM Sodium Citrate with 0.05% Tween-20, pH 6.0) for 12 min, then allowed to cool at room temperature for 30 min. Subsequently, immunohistochemistry was performed. Briefly, slides were washed 3 × 1 min in PBS before exposing them to a blocking solution containing TBST (TBS with 0.1% Tween-20) plus 20% AquaBlock (Cat. No. ab166952, Abcam) for 30 min. Following blocking, slides were incubated overnight at 4°C in primary antibodies (1:500 dilution in blocking solution) for glucagon (Abcam, Cat. No. ab10988, RRID:AB_297642) and insulin (Cat. No. A0564, Dako, Carpinteria, CA). Each slide contained a negative control section incubated only in blocking solution. Slides were next washed three times for 5 min in PBST (PBS with 0.05% Tween-20). Slides were then incubated in the dark for 1 h at room temperature in secondary antibodies (1:500 Goat anti-Guinea Pig, Alexa Fluor 594, Thermo Fisher Scientific, Cat. No. A-11076, RRID:AB_2534120 and Goat Anti-Mouse Alexa Fluor 488, Thermo Fisher Scientific, Cat. No. A-11001, RRID:AB_2534069), washed three times for 5 min in PBST, and coverslipped with ProLong Gold Antifade Mountant with DAPI (Cat. No. P36931, Invitrogen) as the mounting medium. Fluorescent imaging was performed using a Keyence BZ-X710 fluorescence microscope (Keyence America, Itasca, IL) and fluorescent area was quantified with ImageJ software (51).

### Statistical Analyses

To ensure adequacy of sample size, we performed power calculations using PS Software (Power and Sample Size Calculation version 3.1.6). Based on previous studies examining NEFA release from adipose tissue explants taken from fed versus 16-h fasted C57BL6/J mice with α = 0.01, δ = 100, and σ = 25 (44), power calculations showed that at least four mice per experimental group were required to ensure sufficient power to reject the null hypothesis with probability (power) 0.9 (β = 0.9). Statistical analyses were performed in SAS Enterprise Guide 7.1 (SAS Institute Inc., Cary, NC). To assess the effect of genotype within diet group on all dependent variables in our animal studies, we used the mixed model procedure. When statistically significant interactions were found, Tukey’s adjustment for multiple comparisons was used to assess the probability of difference between means. For crossover studies, we conducted paired *t* tests to assess differences between saline and glucagon injections within animals. For ex vivo lipolysis assays, we conducted paired *t* tests to assess differences between control and treatment incubations within each animal. Independent variables were identified as classification variables in all models. Raw data were plotted in GraphPad PRISM Version 8 for Windows (GraphPad Software, San Diego, CA). All data are presented as means ± SE.

## RESULTS

### Glucagon Signaling Does Not Regulate Lipolysis at White Adipose Tissue

**Figure 1.**
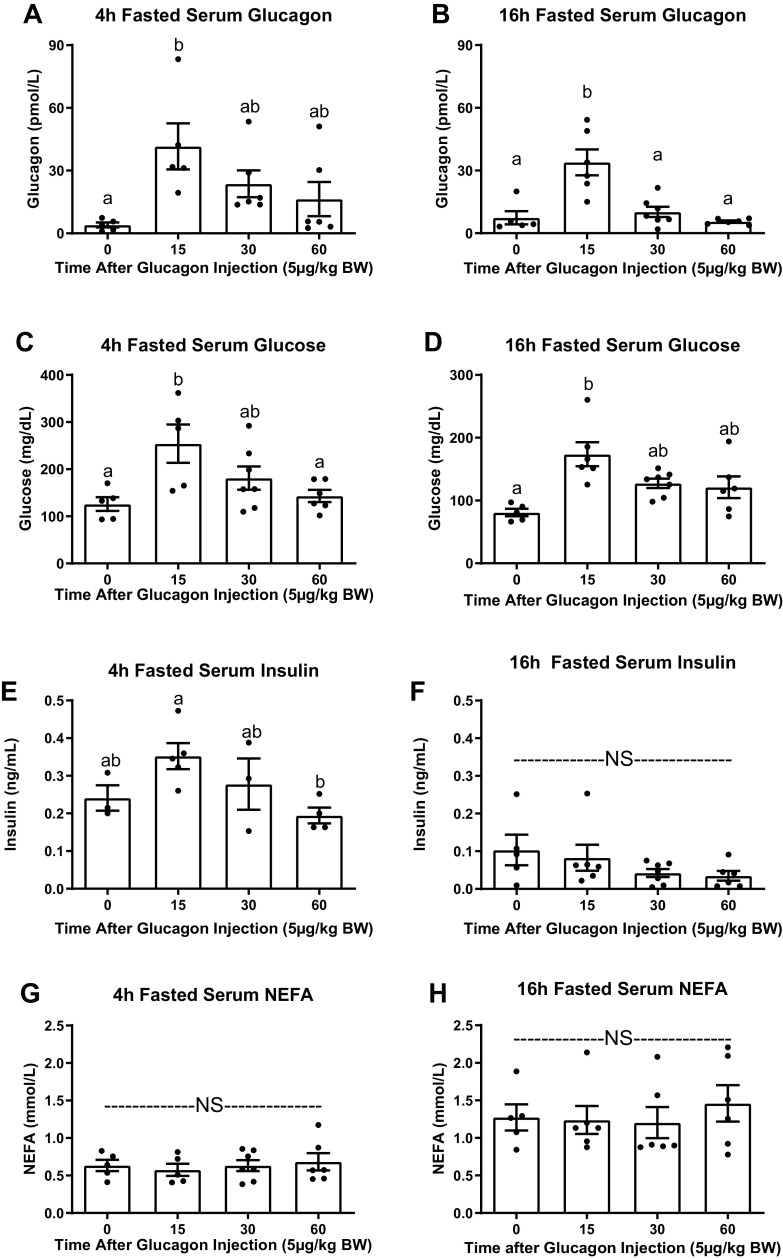
Acute intraperitoneal glucagon administration in wild-type mice. Serum glucagon, glucose, insulin, and NEFA concentrations in wild-type C57BL/6J mice fasted for 4 (*A*, *C*, *E*, and *G*) or 16 (*B*, *D*, *F*, and *H*) h (*n* = 5–7 mice/group for all except 4-h fasted insulin, *n* = 3–5/group). Mice were injected with saline (*time 0*) or glucagon (5 µg/kg) and euthanized at 15, 30, or 60 min after intraperitoneal injection. Data presented as means ± SE. ^a,b^Superscript letters that differ indicate differences within group, *P* < 0.05. One-way ANOVA with Tukey’s adjustment for multiple comparisons. NEFA, nonesterified fatty acids; NS, not significant.

We initially set out to assess the effects of glucagon receptor signaling on lipid homeostasis by examining the effects of acute exogenous glucagon administration on serum NEFA concentration in wild-type C57BL/6J mice. In this time-course study, intraperitoneal glucagon increased serum glucagon in mice fasted for 4 h and 16 h compared with saline-injected mice at 15 min after injection (*P* < 0.05) with a return to levels near baseline by 30 min (Fig. 1, *A* and *B*). Consistent with glucagon’s stimulatory effect on glycogenolysis and gluconeogenesis, intraperitoneal glucagon induced a robust rise in serum glucose in both 4-h and 16-h fasted mice (Fig. 1, *C* and *D*), with a return to basal levels by 30 min after injection. In mice fasted for only 4 h, intraperitoneal glucagon led to a slight, though not statistically significant (*P* = 0.249) rise in serum insulin. At 60 min after glucagon, we observed a significant (*P* = 0.044) decrease in serum insulin compared with 15 min. Acute exogenous glucagon administration did not affect serum insulin in mice fasted for 16 h (Fig. 1, *E* and *F*). Glucagon administration had no effect on serum NEFA concentration in mice, regardless of fasting duration (Fig. 1, *G* and *H*).

**Figure 2.**
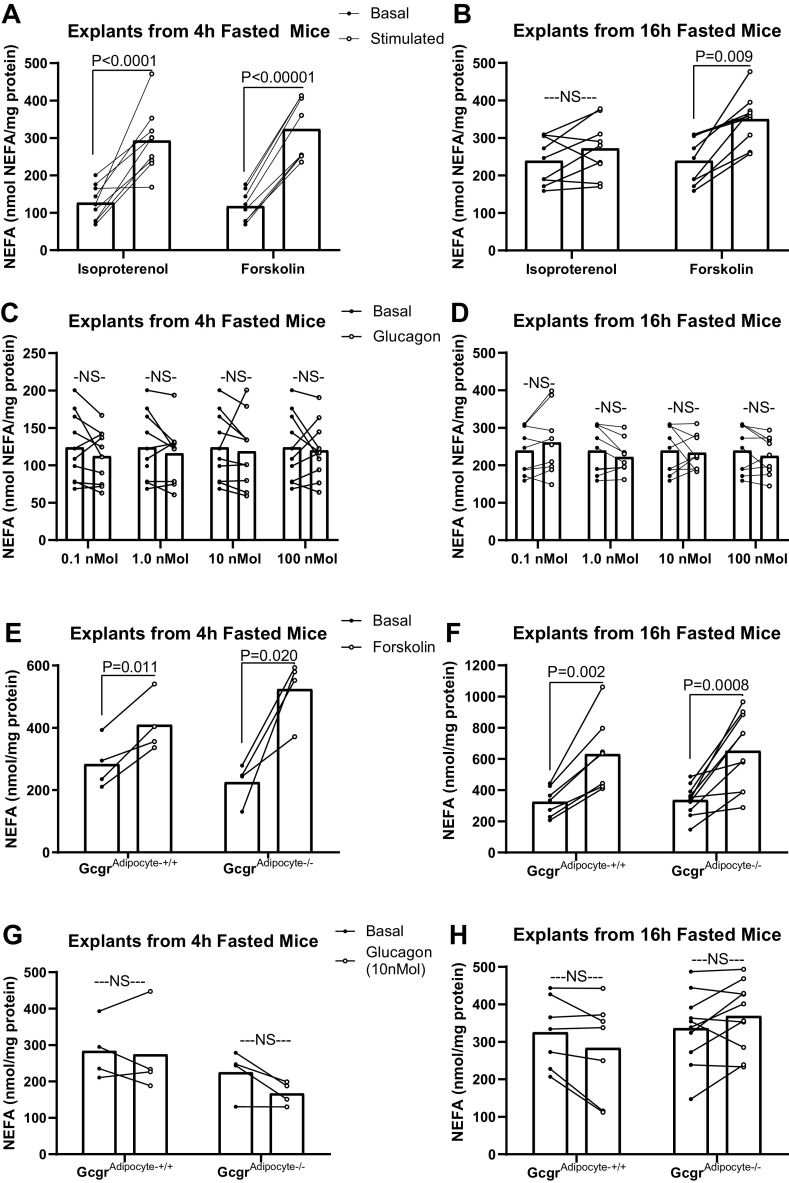
Ex vivo lipolysis from gonadal adipose tissue explants. Explant NEFA release in response to bath application of isoproterenol, forskolin and glucagon was assessed in 4-h and 16-h fasted mice. Isoproterenol and forskolin stimulated explant NEFA release from wild-type C57BL/6J mice fasted for 4 h (*A*; *n* = 10 mice) or 16 h (*B*; *n* = 8). Media NEFA concentrations induced by glucagon in 4 h (*C*; *n* = 10) and 16 h (*D*; *n* = 8) fasted wild-type C57BL/6J mice. NEFA release from explants collected from *Gcgr*^Adipocyte+/+^ vs. *Gcgr*^Adipocyte−/−^ mice fasted for 4 h (*n* = 4) or 16 h (*Gcgr*^adipocyte+/+^: *n* = 7, *Gcgr*^adipocyte−/−^: *n* = 10) and treated with forskolin (*E* and *F*) or glucagon (*G* and *H*). All studies were performed in triplicate explants from each mouse. Data presented as means ± SE; paired samples *t* test. NEFA, nonesterified fatty acids; NS, not significant.

Having demonstrated that exogenous glucagon does not affect circulating NEFA concentration in vivo, we set out to examine the direct effects of glucagon on adipose tissue lipolysis in gonadal adipose tissue explants from mice fasted for either 4 or 16 h. Because it is possible that the observed modest rise in serum insulin in response to exogenous glucagon could suppress adipose tissue lipolysis (Fig. 1*E*), our ex vivo lipolysis assays demonstrated the potential direct effect of glucagon on WAT lipolysis. In explants from 4 h fasted wild-type C57BL/6J mice, isoproterenol, which stimulates lipolysis via activation of both β1 and β2 adrenergic receptors, robustly increased NEFA release (*P* < 0.0001, Fig. 2*A*). In rodents, an extended fast increases lipolysis via catecholamine stimulated β adrenergic signaling and decreases the lipolytic response to isoproterenol (52). Accordingly, isoproterenol did not further stimulate lipolysis in explants from mice fasted for 16 h (Fig. 2*B*). Forskolin, which stimulates lipolysis by directly activating adenylate cyclase and increasing intracellular cyclic AMP concentration, increased NEFA release in explants collected from 4-h and 16-h fasted mice (Fig. 2, *A* and *B*; *P* < 0.01). Insulin (20, 200, and 2,000 nM) robustly decreased lipolysis in explants from 4-h fasted mice (20 nM: *P* = 0.012, 200 nM: *P* < 0.006, 2,000 nM: *P* = 0.009, Supplemental Fig. S1; all Supplemental material is available at https://doi.org/10.6084/m9.figshare.19401233). Consistent with a decrease in insulin’s suppressive action on lipolysis in extended fasting (53), we observed no effect of insulin on NEFA release in explants from 16-h fasted mice (Supplemental Fig. S1). Varying concentrations of glucagon (0.1, 1, 10, and 100 nM) had no effect on NEFA release in explants from mice, regardless of fasting state (Fig. 2, *C* and *D*). We next applied this assay to WAT explants from *Gcgr*^adipocyte+/+^ and *Gcgr*^adipocyte−/−^ mice. We found that glucagon receptor expression at WAT did not affect forskolin-stimulated lipolysis, regardless of fasting state (Fig. 2, *E* and *F*). Similar to our findings in explants from wild-type mice, glucagon did not affect ex vivo lipolysis in explants from *Gcgr*^adipocyte+/+^ or *Gcgr*^adipocyte−/−^ mice, regardless of fasting state (Fig. 2, *G* and *H*).

**Figure 3.**
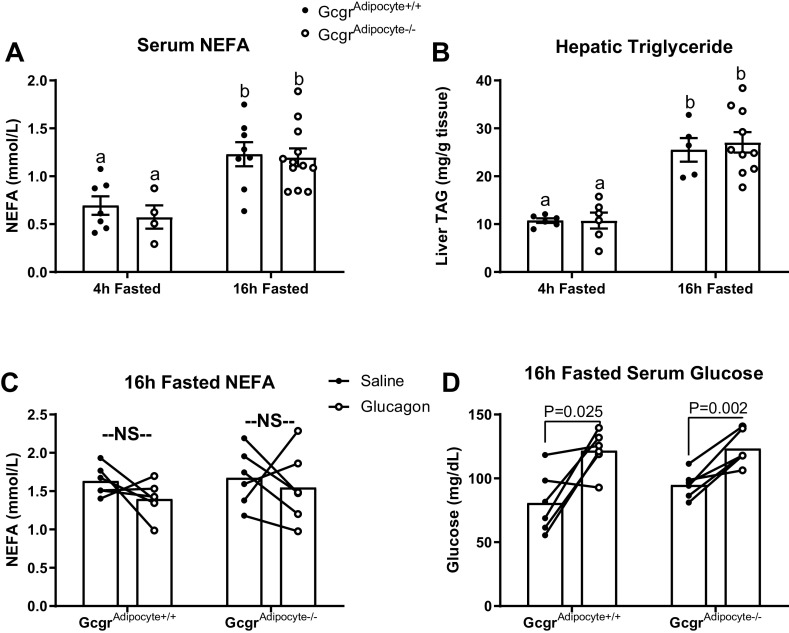
*Gcgr*^adipocyte−/−^ mice fed a low-fat diet have normal fasting-induced changes in serum NEFA concentration and hepatic lipid accumulation. Serum NEFA (*A*) and hepatic triglyceride (*B*) concentrations in mice fasted for 4 h (*n* = 6 or 7 *Gcgr*^adipocyte+/+^ and *n* = 4–6 *Gcgr*^adipocyte−/−^ mice) and 16 h (*n* = 7 *Gcgr*^adipocyte+/+^ and *n* = 10–11 *Gcgr*^adipocyte−/−^). Serum NEFA (*C*) and glucose (*D*) concentrations in 16-h fasted *Gcgr*^adipocyte+/+^ and *Gcgr*^adipocyte−/−^ mice injected with saline and glucagon (*n* = 6/genotype). ^a,b^Superscript letters that differ indicate differences within group, *P* < 0.01. Data presented as means ± SE. *A* and *B*: two-way ANOVA with Tukey’s adjustment for multiple comparisons. *C* and *D*: paired samples *t* test (cross over glucagon responsivity test). NEFA, nonesterified fatty acids; NS, not significant.

Fasting stimulates lipolysis, increases NEFA release, and induces an increase in hepatic triglyceride accumulation (36). Fasting also increases glucagon secretion and signaling (23). To assess if glucagon signaling mediates fasting-induced lipolysis and hepatic lipid accumulation, we examined the effects of an extended fast (16 h) on serum NEFA and hepatic triglyceride concentrations in *Gcgr*^adipocyte+/+^ and *Gcgr*^adipocyte−/−^ mice. Fasting robustly and equally increased both serum NEFA and hepatic triglyceride concentrations in lean *Gcgr*^adipocyte+/+^ and *Gcgr*^adipocyte−/−^ mice (*P* < 0.01, Fig. 3, *A* and *B*). Similarly, diet induced obese *Gcgr*^adipocyte+/+^ and *Gcgr*^adipocyte−/−^ mice responded to a 16 h fast with an equally robust rise in serum NEFA concentration and hepatic triglyceride accumulation (*P* < 0.05, Supplemental Fig. S2, *E* and *F*). Acute exogenous intraperitoneal glucagon did not affect serum NEFA concentration in either *Gcgr*^adipocyte+/+^ or *Gcgr*^adipocyte−/−^ mice (Fig. 3*C*). However, consistent with the gluconeogenic and glycogenolytic actions of glucagon at the liver, intraperitoneal glucagon increased serum glucose, regardless of genotype (*Gcgr*^adipocyte+/+^: *P* = 0.025, *Gcgr*^adipocyte−/−^: *P* = 0.002, Fig. 3*D*).

Both fasting (54) and glucagon (6, 55) increase hepatic FGF21 production via PPARα activation. FGF21 stimulates lipolysis in white adipose tissue (54). We found that both *Gcgr*^adipocyte+/+^ and *Gcgr*^adipocyte−/−^ mice responded to a 16 h fast with an increase in serum FGF21 (*P* < 0.01 for both). Acute intraperitoneal glucagon administration also increased serum FGF21 in both groups 30 min after injection (*P* = 0.03 for *Gcgr*^adipocyte+/+^ and *P* = 0.01 for *Gcgr*^adipocyte−/−^, Supplemental Fig. S3, *A* and *B*).

Circulating NEFA concentration is dependent on the balance between fatty acid release by adipose tissue and clearance by other tissues. To assess the potential role of WAT glucagon receptor signaling on lipid clearance, we performed an oral lipid clearance test in *Gcgr*^adipocyte+/+^ and *Gcgr*^adipocyte−/−^ mice. Lipid clearance after an olive oil oral gavage did not differ between *Gcgr*^adipocyte+/+^ and *Gcgr*^adipocyte−/−^ mice (Supplemental Fig. S4, *A* and *B*). Glucagon decreases hepatic triglyceride secretion (4) and chronic glucagon administration decreases serum cholesterol (33, 34). Intravenous injection of the nonionic detergent, Triton WR1339, inhibits triglyceride hydrolysis by lipoprotein lipase, thereby providing an indication of hepatic triglyceride production. Hepatic triglyceride secretion, as assessed by Triton WR1339, did not differ between *Gcgr*^adipocyte+/+^ and *Gcgr*^adipocyte−/−^ mice (Supplemental Fig. S4*C*), nor did 4-h fasted serum cholesterol in lean (Supplemental Fig. S4*D*) or obese mice (Supplemental Fig. S2*H*).

### Glucagon Signaling at the Adipocyte Does Not Affect Adipokine Release

Leptin stimulates adipose tissue lipolysis (56), whereas adiponectin decreases lipolysis (57). Thus, we explored whether glucagon signaling at the adipocyte regulates the release of these adipokine mediators of lipolysis. *Gcgr*^adipocyte+/+^ and *Gcgr*^adipocyte−/−^ mice responded to a 16 h fast with an equally robust decrease in serum leptin concentration (*P* < 0.01, Supplemental Fig. S5*A*). Acute intraperitoneal glucagon administration did not affect serum leptin or adiponectin concentrations (Supplemental Figs. S5*B* and S6, *A* and *B*) and incubation of adipose tissue explants with glucagon did not affect leptin release into the media (Supplemental Fig. S5*C*).

### Glucagon Signaling at the Adipocyte Does Not Regulate Glucose Homeostasis

**Figure 4.**
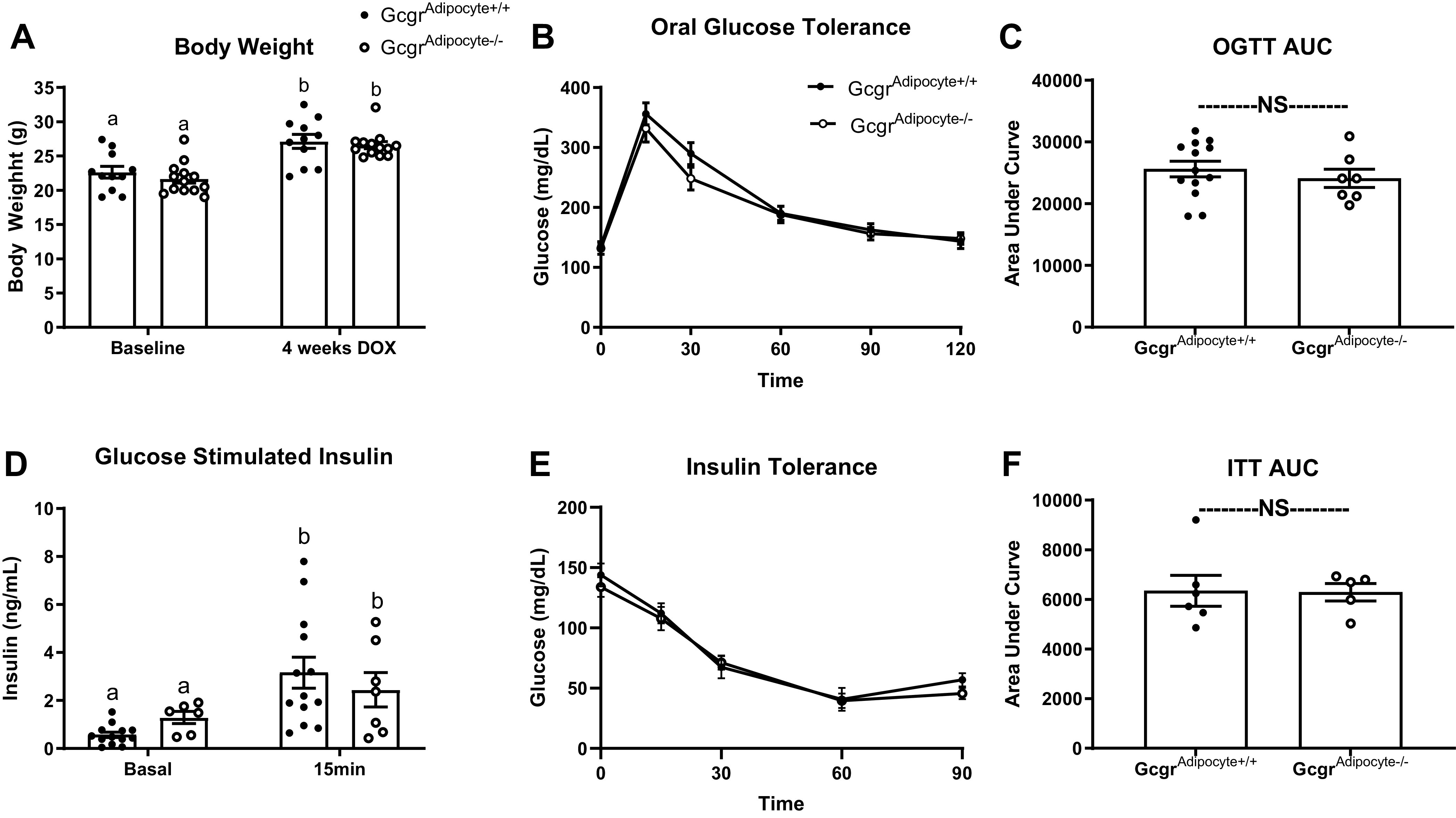
*Gcgr*^adipocyte−/−^ mice fed a low fat diet have a normal response to oral glucose. Body weight (*A*), oral glucose tolerance (OGTT; *B*), OGTT area under the curve (*C*), glucose-stimulated insulin (*D*), insulin tolerance test (ITT; *E*), and ITT area under the curve in lean mice (*F*). For body weight, *n* = 11 *Gcgr*^adipocyte+/+^ and *n* = 13 *Gcgr*^adipocyte−/−^ mice. For OGTT and glucose-stimulated insulin, *n* = 13 *Gcgr*^adipocyte+/+^ and *n* = 7 *Gcgr*^adipocyte−/−^ mice. For ITT, *n* = 6 *Gcgr*^adipocyte+/+^ and *n* = 6 *Gcgr*^adipocyte−/−^ mice). ^a,b^Superscript letters that differ indicate differences within group, *P* < 0.01; two-way ANOVA with Tukey’s adjustment for multiple comparisons for (*A*) body weight and (*D*) glucose stimulated insulin, independent *t* test for (*C*) OGTT and (*F*); ITT AUC. Data presented as means ± SE. AUC, area under the curve; NS, not significant.

Because glucagon receptor signaling in the liver plays a critical role in the maintenance of glucose homeostasis, we set out to determine if glucagon-receptor signaling at the adipocyte affects glucose homeostasis. Body weight (Fig. 4*A*), oral glucose clearance (Fig. 4, *B* and *C*), glucose-stimulated insulin (Fig. 4*D*), and insulin tolerance (Fig. 4, *E* and *F*) did not differ between *Gcgr*^adipocyte+/+^ and *Gcgr*^adipocyte−/−^ mice fed a low-fat diet. Similarly, we observed no differences in diet-induced weight gain, oral glucose clearance, or glucose-stimulated insulin in diet-induced obese *Gcgr*^adipocyte+/+^ and *Gcgr*^adipocyte−/−^ mice (Supplemental Fig. S3, *A*–*D*).

**Figure 5.**
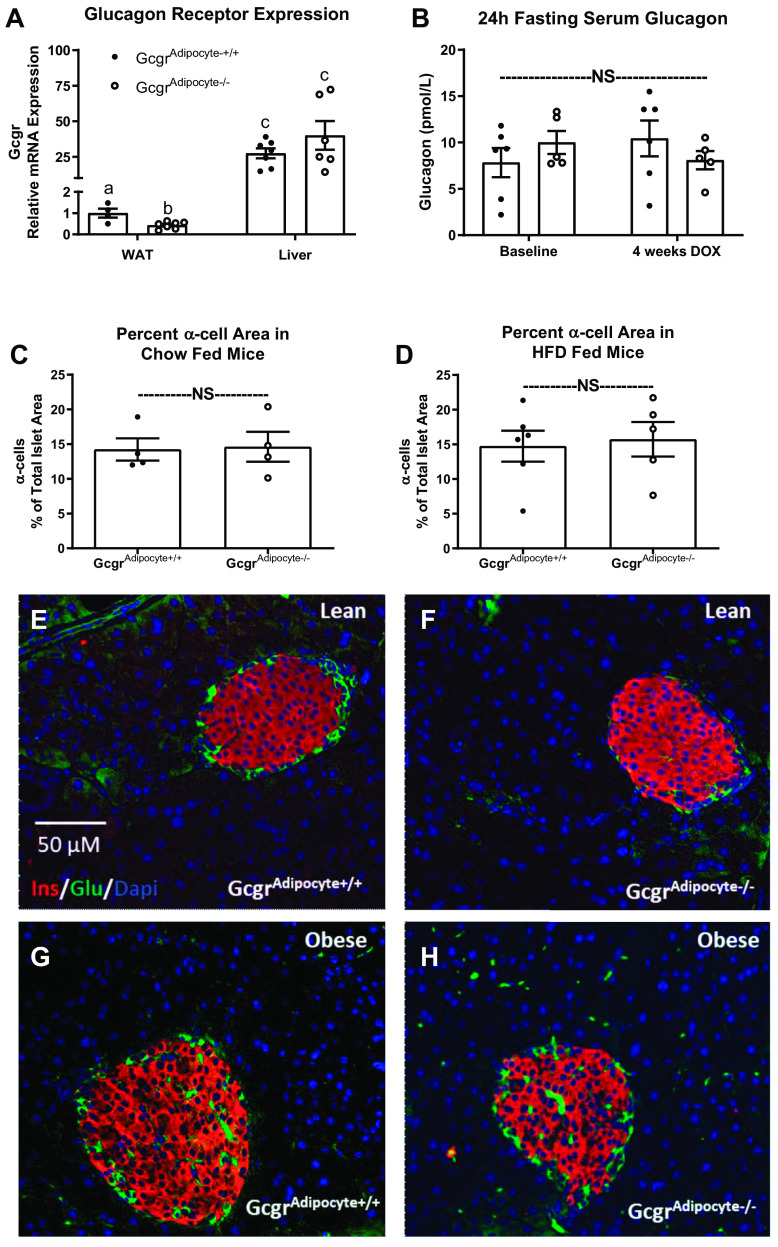
*Gcgr* gene expression, fasting glucagon, and α-cell abundance in *Gcgr*^adipocyte+/+^ and *Gcgr*^adipocyte−/−^ mice. *A*: relative mRNA expression of glucagon receptor in liver (*n* = 7 mice/genotype) and gonadal adipose tissue (*n* = 4 *Gcgr*^adipocyte+/+^ and *n* = 7 *Gcgr*^adipocyte−/−^) in lean mice. *B*: 24-h fasting serum glucagon in lean *Gcgr*^adipocyte+/+^ (*n* = 6) and *Gcgr*^adipocyte−/−^ (*n* = 5) mice before and after 4 wk of doxycycline induction. α-Cell percentage of total islet area in (*C*) lean (*n* = 4/genotype) and (*D*) obese (*n* = 6 *Gcgr*^adipocyte+/+^ and *n* = 5 *Gcgr*^adipocyte−/−^) mice with representative photos (*E*–*H*). ^a,b,c^Superscript letters that differ indicate differences within group, *P* < 0.05; two-way ANOVA with Tukey’s adjustment for multiple comparisons for (*A*) mRNA expression of glucagon receptor and (*B*) fasting serum glucagon, independent *t* test for (*C* and *D*) α-cell percentage of total islet area. Data presented as means ± SE. NS, not significant.

Reflective of glucagon’s critical role in the liver, mice lacking the glucagon receptor at the hepatocyte are hyperglucagonemic with islets that exhibit severe α-cell hyperplasia (1). In islets from both lean and diet-induced obese mice, α-cell abundance (% of total islet area) did not differ between *Gcgr*^adipocyte+/+^ and *Gcgr*^adipocyte−/−^ mice (Fig. 5, *C*–*H*). Furthermore, despite a significant reduction of Gcgr mRNA expression in gonadal WAT, *Gcgr*^adipocyte−/−^ mice had an equally high expression of hepatic *Gcgr* mRNA as *Gcgr*^adipocyte+/+^ mice. Mean *Gcgr* Ct values for adipose tissue from *Gcgr*^adipocyte+/+^ mice was 33 ± 0.65, whereas the mean Ct from *Gcgr*^adipocyte−/−^ mice was 35.12 ± 1.08. In contrast, liver *Gcgr* Ct values were 27.66 ± 0.833 and 27.80 ± 1.14 for *Gcgr*^adipocyte+/+^ and *Gcgr*^adipocyte−/−^ mice, respectively. Thus, glucagon receptor mRNA expression is far lower in adipose tissue than in liver (Fig. 5*A*). Serum glucagon after a 24 h fast did not differ between *Gcgr*^adipocyte+/+^ and *Gcgr*^adipocyte−/−^ mice (Fig. 5*B*). In line with these findings, 16 h of fasting equally increased (*P* < 0.05) hepatic mRNA expression of *Gcgr* and the glucagon-responsive genes: Phosphoenolpyruvate Carboxykinase (*Pck1*), Peroxisome proliferator-activated receptor-α (*Ppara*), and Carnitine Palmitoyl Transferase-1a (*Cpt1a*) in *Gcgr*^adipocyte+/+^ and *Gcgr*^adipocyte−/−^ mice, with no differences between genotype within fasting duration (Supplemental Fig. S7).

### Glucagon Receptor mRNA Is Expressed in Mature Adipocytes

Given the low level of *Gcgr* expression in whole adipose tissue compared with the liver (Fig. 5*A*), we compared the expression of *Gcgr* in isolated mature adipocytes compared with that of the SVF. We found that *Gcgr* mRNA was equally expressed in the enriched adipocyte fraction and SVF (Supplemental Fig. S8*A*). In contrast, the mRNA expression of adipsin, an adipocyte specific marker (58), was ∼90-fold higher in the adipocyte enriched fraction compared with that of SVF (*P* = 0.028, Supplemental Fig. S8*B*).

## DISCUSSION

The potential lipolytic role of glucagon signaling at WAT has long been debated. Glucagon secretion rises in response to an extended fast in lean rodents (23) and humans (59, 60), as does lipolysis (18, 36). Because lipolysis is regulated by G-protein coupled receptors, such as adrenergic receptors, which activate protein kinase A and increase intracellular concentrations of cAMP (61), it has often been assumed that glucagon also mediates fasting-induced WAT lipolysis in a similar fashion through its G-protein coupled receptor. Contrary to this notion, we report that glucagon did not affect WAT lipolysis either indirectly in vivo or through direct action at white adipose tissue ex vivo, regardless of nutritional state. We performed our studies in mice fasted for either 4 or 16 h, employing a 4-h fast as a proxy for the fed state. The 4 h fast ensures that all fed state mice are in a similar nonfasted metabolic state (36, 62, 63). Serum glucagon, insulin, and glucose variability is minimized after a 4 h fast compared with the fed state (23).

Because fasting stimulates adipose tissue lipolysis and the secretion of glucagon, the leading hypothesis is that glucagon stimulates adipose tissue lipolysis. Furthermore, glucagon stimulates intrahepatic lipolysis (64), leading to a rise in circulating NEFA concentration. Our ex vivo lipolysis assays eliminate these confounding factors and clarify that glucagon does not exert direct lipolytic effects on WAT. In our ex vivo lipolysis assays, we utilized forskolin and isoproterenol as positive controls, which stimulate adipose tissue lipolysis. It is important to note that forskolin stimulates lipolysis by directly activating adenylate cyclase and increasing intracellular cyclic AMP concentration. Accordingly, forskolin increased NEFA release in explants collected from 4 and 16 h fasted mice. Yet, isoproterenol only increased NEFA release in explants collected from 4 h fasted mice and did not further stimulate lipolysis in explants from mice fasted for 16 h. This observation is supported by the findings of Giudicelli et al. (52), who showed that fasting decreases the lipolytic response to isoproterenol.

Few studies have fully examined the effects of glucagon on WAT lipolysis in vivo in mice. The majority of studies proposing that glucagon does have a lipolytic effect on WAT were performed on isolated adipocytes from rats (13–17, 65) and humans (9) using supraphysiological levels of glucagon. The generation of mice with a significant reduction of WAT *Gcgr* expression provided a model with which to further explore the physiological role of glucagon receptor signaling at WAT. Using this model, we found that fasting exerted an equally robust increase of serum NEFA concentration and that exogenous glucagon did not affect serum NEFA concentration, regardless of genotype, confirming that glucagon does not exert physiologically relevant effects on adipose tissue lipolysis in vivo. Using this model, we also confirmed that glucagon receptor signaling at WAT does not regulate whole body glucose homeostasis. These studies are in contrast to previous reports that supraphysiological levels of glucagon can increase adipocyte glucose uptake in vitro (9). Finally, applying our ex vivo lipolysis assays to explants from *Gcgr*^adipocyte+/+^ and *Gcgr*^adipocyte−/−^ mice confirmed our finding from experiments in explants from wild-type mice that glucagon does not directly regulate lipolysis in WAT ex vivo. Although Arafat et al. (65) showed that intraperitoneal glucagon administration does increase serum NEFA concentration in vivo in streptozotocin-induced insulin deficient mice, suggesting that glucagon may, in fact, stimulate WAT lipolysis, the authors did not include a healthy nondiabetic group of mice with sufficient insulin levels. Glucagon stimulates intrahepatic lipolysis (64), which is counteracted by insulin (66). Because these studies were only performed in insulinopenic mice, it is difficult to assess if the increase in serum NEFA concentration was due to unregulated hepatic lipolysis as a result of insufficient insulin-mediated suppression of lipolysis or a true increase in adipose tissue lipolysis. Furthermore, the dosage of intraperitoneal glucagon administered in the studies of Arafat et al. (65) (0.05 mg/kg body wt) is supraphysiological. If we estimate that blood represents ∼7% of total body weight (67), this dosage would equate to blood glucagon levels of ∼200 nmol/L. Serum glucagon ranges from <1–7 nmol/L in a lean fed mouse to ∼10–20 nmol/L in a 16–24 h fasted lean mouse and can reach as high as ∼30 nmol/L in an obese insulin-resistant mouse in the fed state (23). We employed a dosage of 5 µg/kg body wt glucagon which, if diluted in blood, could reach a concentration of ∼20 nmol/L, a level that is within physiological glucagon levels, yet still high. Clinical studies showing physiological levels of glucagon do not affect adipose tissue lipolysis in either people with (18) or without (18, 19) diabetes support our findings that glucagon does not regulate WAT lipolysis in mice. In light of our findings, it is important to note that adipocytes are not the major cell type expressing the glucagon receptor within WAT. In fact, through a series of single cell RNA-Seq studies, Campbell and colleagues (10) recently showed that glucagon receptor is predominantly localized to pericytes, with no detectable expression in adipocytes. This was true for both mouse and human WAT. We found that *Gcgr* mRNA was equally expressed in low levels in isolated adipocytes and SVF. Expression of *Gcgr* mRNA in the SVF may explain why our mouse model of adipocyte targeted *Gcgr* knockout only decreased *Gcgr* mRNA by ∼50% in whole WAT.

We recognize that our studies have some limitations. Aberrant glucagon secretion and signaling is a hallmark of both type 2 (insulin resistant) and type 1 (insulin deficient) diabetes. Our studies focused on the role of WAT glucagon receptor signaling in healthy lean mice and diet-induced obese, insulin-resistant mice and our conclusions are limited as such. Glucagon receptor antagonists are in clinical trials as a treatment to lower blood glucose in patients with both type 2 and type 1 diabetes. Thus, further exploration into the potential role of WAT glucagon receptor signaling in type 1 diabetes is required to understand the potential impact of the use of glucagon receptor antagonists in this patient population.

We acknowledge that all experiments performed in this study were conducted in male mice. Women suppress lipolysis in response to insulin more robustly than men (68) and have higher rates of adipose tissue lipolysis during submaximal exercise (69). In line with this observation, female humans (70) and mice (71) respond to epinephrine with greater rates of lipolysis compared with males. Altogether, the observed sex differences in the suppression of adipose tissue lipolysis warrant additional studies examining potential sex differences in the lipolytic response to glucagon receptor signaling.

Finally, our studies address the effects of acute glucagon action on WAT lipid homeostasis. Future studies should evaluate the potential chronic effects of glucagon signaling on WAT lipid metabolism. To assess the site of action, these studies will require the use of adipocyte- and hepatocyte-specific *Gcgr* knockout mice.

### Conclusions

People with obesity and overweight constitute 1/3 and 2/3 of the US population, respectively (72). Given emerging evidence that di- and triagonists that include glucagon receptor agonists are effective in treating obesity and dyslipidemia in rodents (73–75), nonhuman primates (76), and humans (77, 78), it is critical to understand the tissue-specific effects of glucagon receptor action. We have established that physiological levels of glucagon do not regulate WAT lipolysis, either directly or indirectly. Furthermore, our studies show that glucagon receptor signaling at WAT does not affect whole body lipid or glucose homeostasis. In concurrence with others (11, 18–20), our studies suggest that the metabolic effects of glucagon receptor agonism are not mediated through action at white adipose tissue.

## SUPPLEMENTAL DATA

10.6084/m9.figshare.19401233 Supplemental Figs. S1–S8: https://doi.org/10.6084/m9.figshare.19401233.

## GRANTS

This work was supported by the National Institutes of Health Grants F32-DK107058, K99-AG055649, and R00-AG055649 (to J.H.S.). This study was also supported by Diabetes Canada (to J.L.B.).

## DISCLOSURES

No conflicts of interest, financial or otherwise, are declared by the authors.

## AUTHOR CONTRIBUTIONS

J.L.B. and J.H.S. conceived and designed research; A.V., T.M., and J.H.S. performed experiments; A.V., T.M., and J.H.S. analyzed data; J.H.S. interpreted results of experiments; A.V. and J.H.S. prepared figures; J.H.S. drafted manuscript; J.L.B. edited and revised manuscript; A.V., T.M., J.L.B., and J.H.S. approved final version of manuscript.
